# DIGITAL DIVIDE AND DIFFICULTIES IN ACQUIRING HEALTH RESOURCES IN DISABLED OLDER ADULTS DURING THE PANDEMIC

**DOI:** 10.1093/geroni/igac059.2876

**Published:** 2022-12-20

**Authors:** Eunjin Yang, Min Jung Kim, Kyung Hee Lee

**Affiliations:** Yonsei University College of Nursing, Seoul, Seoul-t’ukpyolsi, Republic of Korea; College of Nursing, Yonsei University, Seoul, Seoul-t’ukpyolsi, Republic of Korea; Yonsei University, Seoul, Seoul-t’ukpyolsi, Republic of Korea

## Abstract

The digital divide refers to the gap between those who are connected and those who are not connected to the internet and technologies. Although COVID-19 worsens the digital divide and health inequality, few studies have focused on the relationship between digital divide and difficulties in acquiring of health resources during the pandemic. This study aimed to identify the relationship between internet use and difficulties in acquiring health resources among older adults with disabilities during the COVID-19 pandemic. This secondary analysis study included 4,871 older adults aged 55 and older among 7,025 total responders of the 2020 survey of people with disabilities in South Korea. As the results, only 23.7% of older adults with disabilities used the internet. Non-internet users are more likely to have difficulties in acquiring COVID-19-related information (aOR 1.59) and buying and using personal protective equipment (aOR 1.36). However, difficulties in using medical services (aOR 1.21) was not statistically significant. Considering that older adults with disabilities have triple burdens from old age, disabilities, and the digital divide amid COVID-19, healthcare providers need to pay more attention to mitigate gaps between internet users and non-non users among this population. By narrowing the digital divide, decreasing health gaps and increasing well-being among older adults with disabilities will be guaranteed.

